# Supplementary material for: Preventing spread of aerosolized infectious particles during medical procedures: A lab-based analysis of an inexpensive plastic enclosure
Source: PLoS One. 2022 Sep 22;17(9):e0273194. doi: 10.1371/journal.pone.0273194 (PMC9499281; doi:10.1371/journal.pone.0273194)
Supplement: S1 Appendix — (DOCX) [file pone.0273194.s001.docx]

S1 Appendix. Further discussion on covers, particle generation, instruments, and other considerations.

Desirable properties in the plastics used for covers

All plastics tested used the same base composition for the material, a low-density polyethylene. The primary differences between the materials were the thickness, adhesives, and opacity. Thicker, non-opaque, materials were found to perform better, as they were more resistant to tearing around the wrists and easier to operate with. Self-adhesive properties are desirable, and if strong enough, could preclude the use of tape or other sealing methods. These are not a requirement for good performance. The secondary drape material is less important as it is not subject to stress. However, thicker material and/or adhesives can provide a better seal over the hand holes in the base layer. Fabrication costs for the enclosure was $300 per unit. This cost is expected to be variable depending on manufacturer and size of order placed. Suggested moving wrap can be obtained for less than $40 (USD) to cover 41 containers. 3M Steri-Drapes can be found for $100 per 40, and $120 per 12 upper body blankets, with 0.5 used per enclosure use. This puts costs of disposables at less than $10 per use of the enclosure.

# **Aerosol particle composition**

Aerosol particles were generated primarily from a 1 mg/mL bulk solution of aqueous ammonium sulfate (Sigma-Aldrich, ACS grade). In addition, particle composition was varied to better mimic organic components in cough droplets using a mixed solution of 2 mg/mL azelaic acid (Acros Organics, 98%) and 10 mg/mL sucrose (Sigma, ACS grade) in deionized water. While most of the relevant aerosol behavior is driven by particle size, composition can potentially influence the electrical properties of the particles (e.g., dielectric constant and polarizability). Hygroscopicity can also influence aerosol behavior. No noticeable difference was observed in particle trapping efficiencies with different particle compositions. Fluorescein salt (Sigma-Aldrich) was used in trace amounts for fluorescent experiments in a 1 mg/mL ammonium-sulfate solution.

# **Instrumentation description and capabilities**

Aerosol measurements were obtained with two condensation particle counters (CPCs, TSI, 3772 and 3775) for total number concentrations, an aerosol monitor (TSI, DustTrak DRX 8533) for aerosol volume/mass size distributions, and an aerodynamic particle sizer (TSI, 3321) for aerosol number size distributions. The CPCs have a 50% diameter cutoff at 10 nm (model 3772) and 4 nm (model 3775). The CPCs therefore measured all aerosol particles present with integration time either 1 or 10 seconds. CPC 3775 was chosen to operate inside the enclosure due to its lower flow rate and ability to measure a higher total number concentration of particles (up to 10^7^ cm^-3^). To determine the particle size distribution for each particle generation method, the CPCs were combined with a differential mobility analyzer to produce a scanning mobility particle sizer (SMPS).

The DustTrak measured particles between 0.1 μm to 15 μm in aerodynamic diameter. The APS can nominally detect particles between 0.3 μm to 20 μm, with accurate sizing of particles greater than 0.52 μm in aerodynamic number and mass diameter. This specific APS instrument is known to undercount particles below 1 micron, based on a recent manufacturer calibration (due to low scattering laser power). As such, when comparing concentrations in the size region in which the SMPS and the APS overlap, the SMPS should be considered the more accurate measure [1]. The placement of the DustTrak or APS inside the enclosure depended on the test; generally, the DustTrak was used inside due to its ability to handle higher aerosol mass loadings, and the APS was used outside due to its sensitivity at lower aerosol number concentrations.

The CPCs measured number concentrations, the DustTrak reported volume/mass concentrations, and the APS reported both. The size range of the CPCs are wider and have near identical size detection capabilities between the two models. Thus, considering the SMPS’s long integration time, the APS’s known undercounting of submicron particles, the OPC’s unique size range and large bins (PM_10_, PM_2.5_, PM_1_ and total), we defaulted to the CPC measurements for all figures and data shown in paper, apart from S1 and S2. As such, all aerosol measurements are presented in number concentration (number per cm^3^ air) or normalized percentages from a ratio of number concentrations.

# **Aerosol Sampling**

Background particle concentrations were 500-2000 per cm^3^ depending on day. Peak internal concentrations varied by nebulizer and enclosure setup but ranged from 5000-100000 per cm^3^. The upper range of this likely experienced under counting issues due to overlapping particle “coincidence” detection events in the CPCs for concentrations > 10^5^ cm^3^.

The lab testing was conducted in the Air Quality Laboratory at Carnegie Mellon University in Pittsburgh, PA during the spring and summer months. Environmental HVAC controls kept general lab RH and temperature fairly consistent throughout experimentation. Sampling lines were located on the level of enclosure areas being measured: approximately 32 cm above table level for front and side ports, 1-2 cm above table level for patient entrance. The enclosure was placed on top of a thick layer of fabric to act as a surrogate for a hospital bed.

A control experiment with no enclosure was not possible in the available lab space. Permanent exhaust lines/snorkels in the lab ensure a constant directional air flow in the lab. Nebulized particles completely open to room air followed this flow. A similar situation would be present in any indoor environment with active ventilation and/or active personnel, such as a hospital setting. The enclosure without coverings configuration was enough to disrupt this ambient air flow and sampling lines were placed ‘downwind’ of any opening relative to the prevailing air flow in the laboratory. The measurements as a function of distance away from the enclosure (made as close as 7.6 cm) provide a good assessment of the number fraction that escapes from inside the enclosure, and how quickly these aerosols become entrained into the background air with its much lower background aerosol concentration < 2000 cm^3^.

# **Note on sources of particle loss**

Particle loss rates are largely dictated by particle size. The suggested 15–20-minute timeframe is enough for all particles 5 µm and larger to gravitationally settle within the enclosure. 50-90% of particles in the 3-5 µm range should also have time to settle within this time frame. Particles on the smaller end are lost primarily through coagulation with larger particles and wall loss due to diffusion and electrostatic attraction. The intermediary sized particles (300–2000 nm) are generally the hardest to account for. Some of them will be lost via the various mechanisms described above, and some will be lost due to leakage out of the openings and gaps in the enclosure. Suction of air inside the enclosure will be the most reliable method for fast and near total removal of these particles, such as using a vacuum line inserted through the side hole of the enclosure. However, even without suction, the enclosure will still reduce net exposure to all particle sizes of aerosol produced inside the enclosure in the outside environment surrounding the enclosure.

# **Application of suction**

Suction in the enclosure was produced in some experiments by inserting a vacuum hose (3/8” o.d.) through the side hole and pulling flow sufficiently away from the sampling lines and nebulizer. The suction line was near the wall of the enclosure to not interfere with the nebulizer. Either a 15 LPM or 30 LPM suction was applied, with a suction canister in line. Much above 30 LPM and the canister would collapse. This setup is typically used in the operating room to remove fluids aspirated from the patient. A small HEPA filter was used inline that did not impede the rate of flow. During experiments looking at the impacts of applied suction, sampling instruments removed 0.3 LPM. When suction was not being considered an experimental variable, the instruments sampled at 3.3 LPM total.

# **Statistical analysis**

Cursory statistical analysis was conducted in Microsoft Excel 365. Most statistical analysis was conducted in MATLAB 2019a using built in functions. One exception was the Games-Howell test code which can be found at:

Trujillo-Ortiz, A. and R. Hernandez-Walls. (2003) http://www.mathworks.com/matlabcentral/fileexchange/loadFile.do?objectId=3676&objectType=FILE

One edit was made to this code, where the output of line 168 was divided by sqrt(2) to correct an error in the code.

Of note is the difference in use between parametric and nonparametric tests. The wide difference in consistency between data collected inside the enclosure, close to the enclosure, and far from the enclosure made consistent use of exact statistical testing parameters difficult. In general, data collected within the enclosure and near the enclosure was widely reproducible and generally followed assumptions associated with parametric testing. Dilution and the rapid dispersion of aerosol once outside the enclosure are the root drivers behind the differences in the degree of variability of the different aerosol measurement locations.

# References

1. Knopf, D. A., Barry, K. R., Brubaker, T. A., Jahl, L. G., Jankowski, K. A., Li, J., Lu, Y., Monroe, L. W., Moore, K. A., Rivera-Adorno, F. A., Sauceda, K. A., Shi, Y., Tomlin, J. M., Vepuri, H. S. K., Wang, P., Lata, N. N., Levin, E. J. T., Creamean, J. M., Hill, T. C. J., … Liu, X. (2021). Aerosol–Ice Formation Closure: A Southern Great Plains Field Campaign. *Bulletin of the American Meteorological Society*, *102*(10), E1952–E1971. https://doi.org/10.1175/BAMS-D-20-0151.1
