# Supplementary material for: Preventing spread of aerosolized infectious particles during medical procedures: A lab-based analysis of an inexpensive plastic enclosure
Source: PLoS One. 2022 Sep 22;17(9):e0273194. doi: 10.1371/journal.pone.0273194 (PMC9499281; doi:10.1371/journal.pone.0273194)
Supplement: S1 Table — (DOCX) [file pone.0273194.s002.docx]

# **S2 Table. Summary of statistical result for cover comparison**

| **Comparison Between Cover Configurations** | | |
| --- | --- | --- |
| Configuration | | Reduction in peak inside concentration (%) |
| 1 | No plastic cover on enclosure openings | 30 ± 10% |
| 2 | 1-layer over opening (industrial furniture wrap) | 86 ± 6% |
| 3 | 2-layers over opening (industrial furniture wrap + 3M steri-drape) | 97 ± 3% |
| 4 | 2-layer over opening (Press ‘n Seal + 3M steri-drape_ | 99 ± 1% |
| 5 | 2-layers over opening and 15 LPM suction (industrial furniture wrap + 3M steri-drape) | 98 ± 3% |

P-value for one-way ANOVA = 7.89 E-6

| **Games-Howell Results from Cover Comparison Tests** | | | | | |
| --- | --- | --- | --- | --- | --- |
| Config 1 | Config 2 | Mean Diff. | Crit value | Deg Free | Conclusion |
| 1 | 2 | 15.94 | 8.4 | 6 | S |
| 1 | 3 | 27.0 | 16.3 | 6 | S |
| 1 | 4 | 29.4 | 19.1 | 4 | S |
| 1 | 5 | 28.4 | 16.7 | 6 | S |
| 2 | 3 | 11.1 | 7.8 | 5 | S |
| 2 | 4 | 13.5 | 10.4 | 3 | S |
| 2 | 5 | 12.5 | 8.4 | 5 | S |
| 3 | 4 | 2.4 | 2.7 | 6 | NS |
| 3 | 5 | 1.4 | 1.2 | 7 | NS |
| 4 | 5 | 1.0 | 1.0 | 6 | NS |

Where the numbers in the ‘Config’ columns correspond to configurations in previous table. S = Significant difference, NS = Non-significant difference
