# Supplementary material for: Preventing spread of aerosolized infectious particles during medical procedures: A lab-based analysis of an inexpensive plastic enclosure
Source: PLoS One. 2022 Sep 22;17(9):e0273194. doi: 10.1371/journal.pone.0273194 (PMC9499281; doi:10.1371/journal.pone.0273194)
Supplement: S2 Table — (DOCX) [file pone.0273194.s003.docx]

# **S3 Table. Complete survey results of medical professionals that had used the enclosure for simulated intubation/extubation procedures on mannequins at two different hospitals**

^a^Type of medical professional:

AGH: Allegheny General Hospital

WPH: West Penn Hospital

CRNA: Certified Registered Nurse Anesthetist

MD: Medical Doctor

Scale:

| **Strongly Agree Agree Neutral Disagree Strongly Disagree** | | | | | | | | | | | | | |
| --- | --- | --- | --- | --- | --- | --- | --- | --- | --- | --- | --- | --- | --- |
| *5 4 3 2 1* | | | | | | | | | | | |  | |
| **Q1.       This box permits me to extubate appropriate sized patients safely.** | | | | | | | | | | | | | |
|  | | **Response** | **5** | | **4** | | **3** | | | **2** | | **1** | |
|  | | AGH (n = 20) | 10 (50%) | | 9 (45%) | | 1 (5%) | | | 0 | | 0 | |
|  | | WPH (n =19) | 13 (68%) | | 6 (32%) | | 0 | | | 0 | | 0 | |
|  | | CRNA (n = 29) | 16 (55%) | | 12 (41%) | | 1 (3%) | | | 0 | | 0 | |
|  | | MD (n = 10) | 7 (70%) | | 3 (30%) | | 0 | | | 0 | | 0 | |
|  | | **Total (n=39)** | **23 (59%)** | | **15 (38%)** | | **1 (3%)** | | | **0** | | **0** | |
| **Q2.       I can place and access all needed airway equipment easily.** | | | | | | | | | | | | |  |
|  |  | | |  | |  | |  |  | |  | |  |
|  | **Response** | | | **5** | | **4** | | **3** | **2** | | **1** | |  |
|  | AGH (n = 20) | | | 5 (25%) | | 15 (75%) | | 0 | 0 | | 0 | |  |
|  | WPH (n =19) | | | 13 (68%) | | 6 (32%) | | 0 | 0 | | 0 | |  |
|  | CRNA (n = 29) | | | 18 (62%) | | 11 (38%) | | 0 | 0 | | 0 | |  |
|  | MD (n = 10) | | | 7 (70%) | | 3 (30%) | | 0 | 0 | | 0 | |  |
|  | **Total (n=39)** | | | 18 (46%) | | 21 (54%) | | 0 | 0 | | 0 | |  |

| **Q3.       I can see the mannequin and procedural field clearly.** | | | | | |  |
| --- | --- | --- | --- | --- | --- | --- |
|  |  |  |  |  |  |  |
|  | **Response** | **5** | **4** | **3** | **2** | **1** |
|  | AGH (n = 20) | 14 (70%) | 6 (30%) | 0 | 0 | 0 |
|  | WPH (n =19) | 15 (79%) | 3 (16%) | 1 (5%) | 0 | 0 |
|  | CRNA (n = 29) | 20 (69%) | 8 (28%) | 1 (3%) | 0 | 0 |
|  | MD (n = 10) | 9 (90%) | 1 (10%) | 0 | 0 | 0 |
|  | **Total (n=39)** | 29 (74%) | 9 (23%) | 1 (3%) | 0 | 0 |

| **Q4.       The box ergonomics are acceptable.** | | | |  |  |  |
| --- | --- | --- | --- | --- | --- | --- |
|  |  |  |  |  |  |  |
|  | **Response** | **5** | **4** | **3** | **2** | **1** |
|  | AGH (n = 20) | 7 (35%) | 11 (55%) | 1 (5%) | 1 (5%) | 0 |
|  | WPH (n =19) | 13 (68%) | 6 (32%) | 0 | 0 | 0 |
|  | CRNA (n = 29) | 14 (48%) | 14 (48%) | 0 | 1 (3%) | 0 |
|  | MD (n = 10) | 6 (60%) | 3 (30%) | 1 (10%) | 0 | 0 |
|  | **Total (n=39)** | 20 (51%) | 17 (43%) | 1 (3%) | 1 (3%) | 0 |

| **Q5.       A helper can provide effective assistance in using the box.** | | | | | | |
| --- | --- | --- | --- | --- | --- | --- |
|  |  |  |  |  |  |  |
|  | **Response** | **5** | **4** | **3** | **2** | **1** |
|  | AGH (n = 20) | 10 (50%) | 9 (45%) | 1 (5%) | 0 | 0 |
|  | WPH (n =19) | 15 (79%) | 4 (21%) | 0 | 0 | 0 |
|  | CRNA (n = 29) | 17 (59%) | 11 (38%) | 1 (3%) | 0 | 0 |
|  | MD (n = 10) | 8 (80%) | 2 (20%) | 0 | 0 | 0 |
|  | **Total (n=39)** | 25 (64%) | 13 (33%) | 1 (3%) | 0 | 0 |

| **Q6.       The box permits me to safely perform endotracheal suctioning.** | | | | | | |
| --- | --- | --- | --- | --- | --- | --- |
|  |  |  |  |  |  |  |
|  | **Response** | **5** | **4** | **3** | **2** | **1** |
|  | AGH (n = 20) | 10 (50%) | 8 (40%) | 2 (10%) | 0 | 0 |
|  | WPH (n =19) | 12 (63%) | 6 (32%) | 1 (5%) | 0 | 0 |
|  | CRNA (n = 29) | 16 (55%) | 10 (34%) | 3 (10%) | 0 | 0 |
|  | MD (n = 10) | 6 (60%) | 4 (40%) | 0 | 0 | 0 |
|  | **Total (n=39)** | 22 (56%) | 14 (36%) | 3 (8%) | 0 | 0 |
