# Supplementary material for: Preventing spread of aerosolized infectious particles during medical procedures: A lab-based analysis of an inexpensive plastic enclosure
Source: PLoS One. 2022 Sep 22;17(9):e0273194. doi: 10.1371/journal.pone.0273194 (PMC9499281; doi:10.1371/journal.pone.0273194)
Supplement: S1 Fig — (DOCX) [file pone.0273194.s004.docx]

**
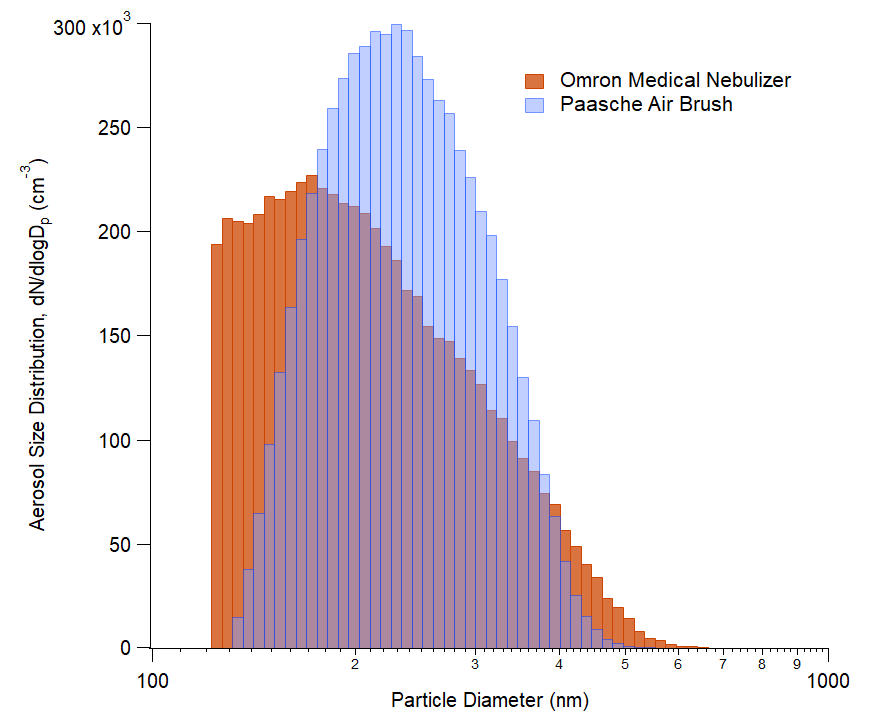
****S4 Figure**. **Small particle size distribution from nebulizers.** Number size distributions of particles between 0.12 µm and 0.6 µm generated by the Paasche airbrush (blue) and Omron medical nebulizer (orange) measured with the scanning mobility particle sizer (SMPS). Particles below 120 nm were not measured to shorten measuring time to limit the impact of the initial rapid particle decay. It is also presumed that viral particles would not be able to be present in particles below this size. The airbrush has a mode at 0.23 µm and the medical nebulizer at 0.17 µm.

**
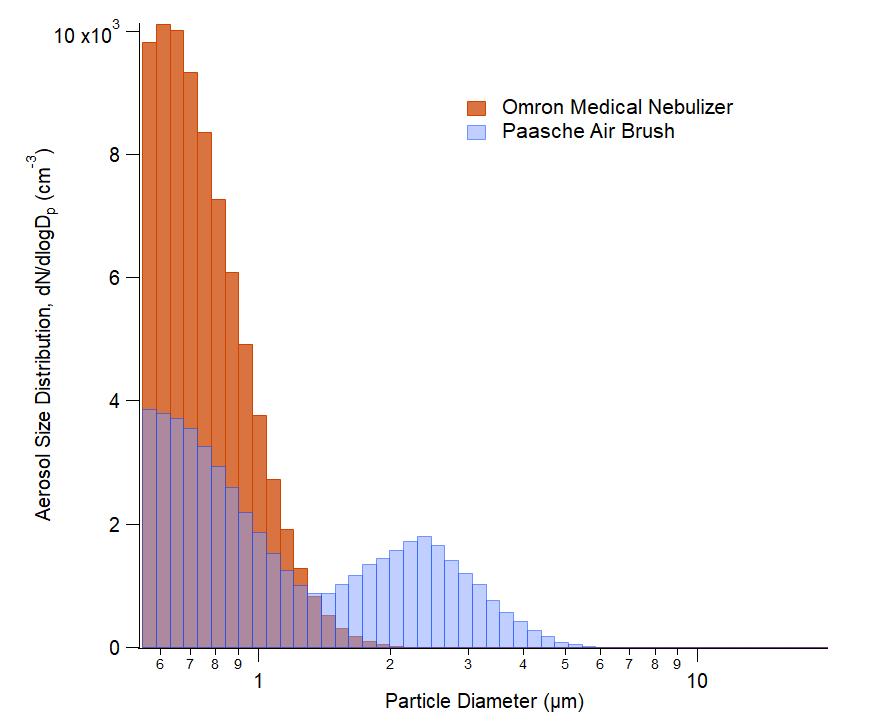
**

**S4 Figure**. Number size distribution measured with the Aerodynamic Particle Sizer (APS) of particles >0.5 µm generated from the Paasche Airbrush (blue) and Omron medical nebulizer (orange). The airbrush has 2 modes, one at 0.6 µm and another at 2.1 µm. The second mode is likely the result of decreasing counting efficiency of the APS in the lower size range. The medical nebulizer has one at 0.7 µm.
