# Supplementary material for: Preventing spread of aerosolized infectious particles during medical procedures: A lab-based analysis of an inexpensive plastic enclosure
Source: PLoS One. 2022 Sep 22;17(9):e0273194. doi: 10.1371/journal.pone.0273194 (PMC9499281; doi:10.1371/journal.pone.0273194)
Supplement: S2 Fig — (DOCX) [file pone.0273194.s005.docx]

**
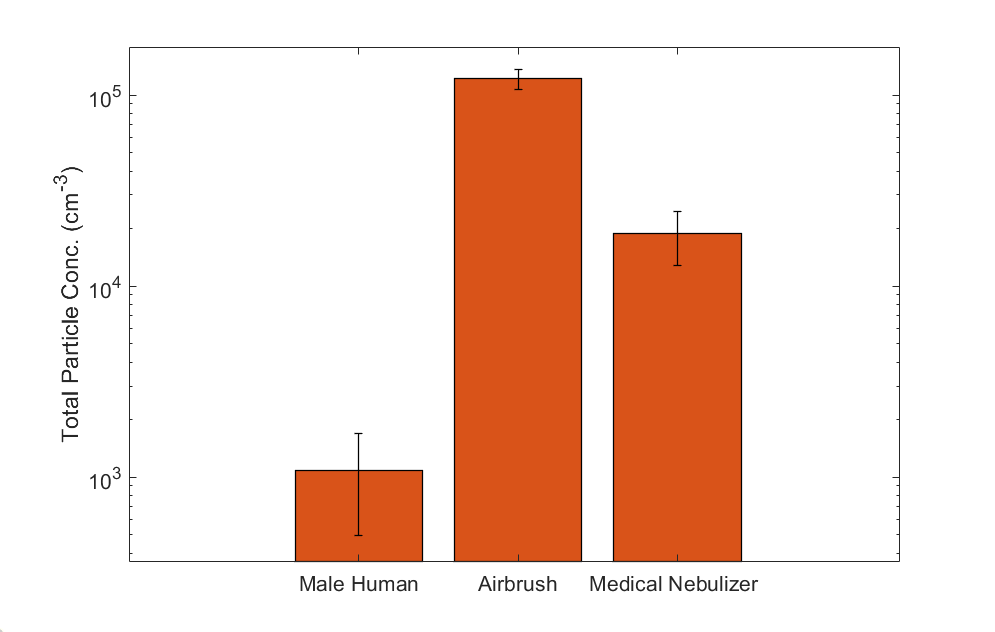
****S5 Figure**. **Comparison of total particle concentration generated from real and simulated coughs**. The average male human cough (Yang et. al, 2007) compared with the simulated Paasche airbrush cough and an extended burst from the Omron medical nebulizer. The medical nebulizer and airbrush means are derived from the peak concentration achieved within the uncovered enclosure.
