# Supplementary material for: Preventing spread of aerosolized infectious particles during medical procedures: A lab-based analysis of an inexpensive plastic enclosure
Source: PLoS One. 2022 Sep 22;17(9):e0273194. doi: 10.1371/journal.pone.0273194 (PMC9499281; doi:10.1371/journal.pone.0273194)
Supplement: S3 Fig — (DOCX) [file pone.0273194.s006.docx]

**
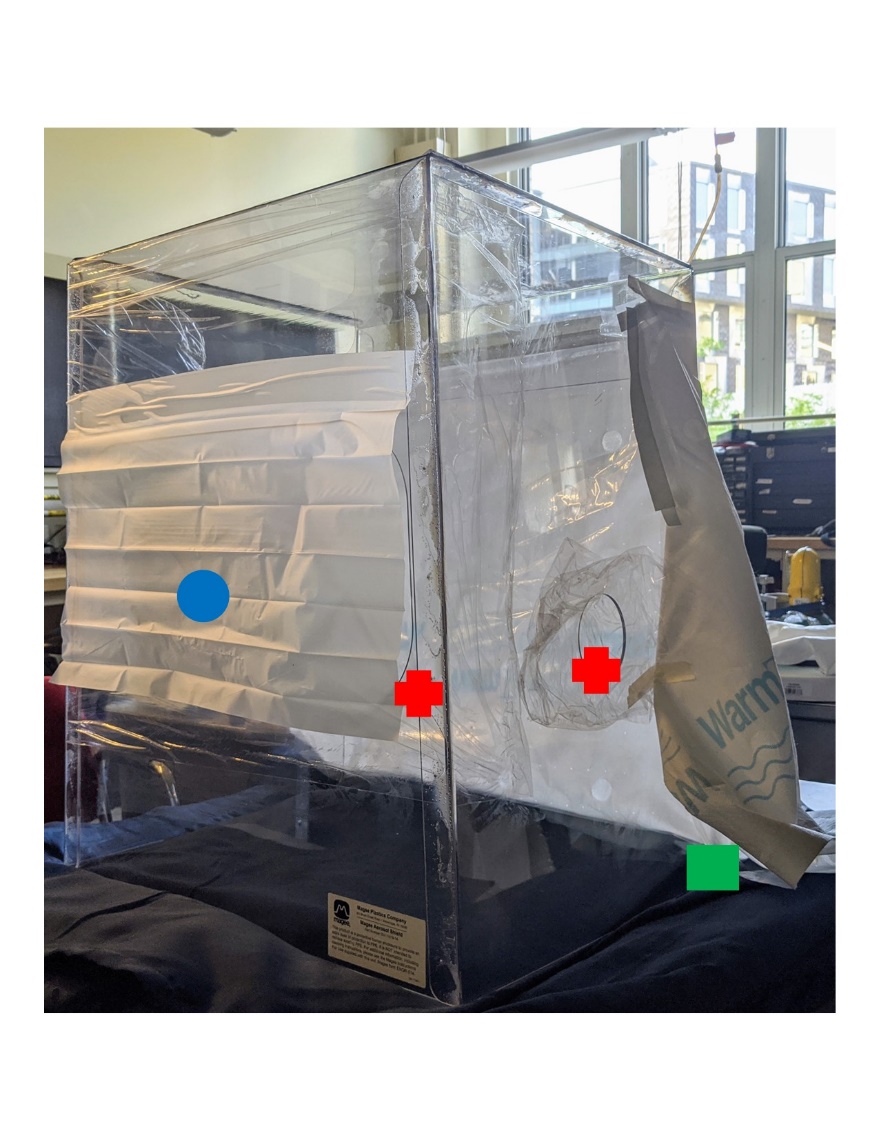
**

**S6 Figure. Sampling intake locations for enclosure.** External aerosol sampling locations near the enclosure indicated by a marker. Blue circle indicates sampling location when measuring in the no covers or one cover with hand slits configuration. The red crosses indicate locations used for dual cover configuration measurements. The green square indicates other sampling locations tested near the side hole and further away from the enclosure. All internal sampling lines and vacuum hose were inserted through the side port. Nebulizers were placed at the center of the enclosure and pointed 70-90º above horizontal table level between 12-17 cm above the bottom surface, depending on which type of nebulizer.
