# Supplementary material for: Preventing spread of aerosolized infectious particles during medical procedures: A lab-based analysis of an inexpensive plastic enclosure
Source: PLoS One. 2022 Sep 22;17(9):e0273194. doi: 10.1371/journal.pone.0273194 (PMC9499281; doi:10.1371/journal.pone.0273194)
Supplement: S4 Fig — (DOCX) [file pone.0273194.s007.docx]

**
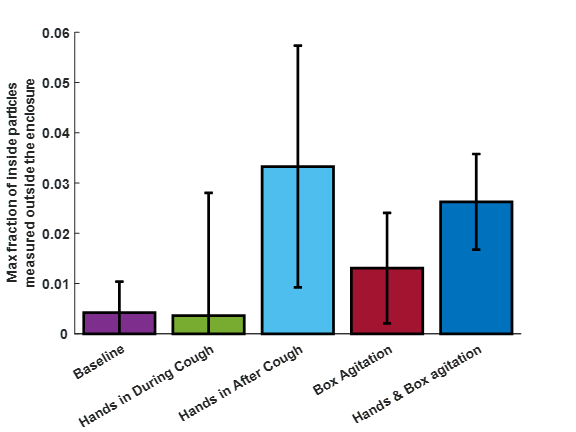
**

**S7 Figure. Comparison of fraction of particle escape during enclosure agitation.** Different results of the agitation experiments with error bars representing standard deviation between 3 trials. The 2-layer furniture wrap and Steri-Drape were used for these experiments, with hands covered in nitrile gloves. Baseline is the 2-layer covered enclosure with no hands or jostling. No statistical difference was found with one-way ANOVA. Jostling and inserting/removing hands in the box will not lead to large increases in particle concentrations outside of the box.
