# Supplementary material for: Preventing spread of aerosolized infectious particles during medical procedures: A lab-based analysis of an inexpensive plastic enclosure
Source: PLoS One. 2022 Sep 22;17(9):e0273194. doi: 10.1371/journal.pone.0273194 (PMC9499281; doi:10.1371/journal.pone.0273194)
Supplement: S5 Fig — (DOCX) [file pone.0273194.s008.docx]

**
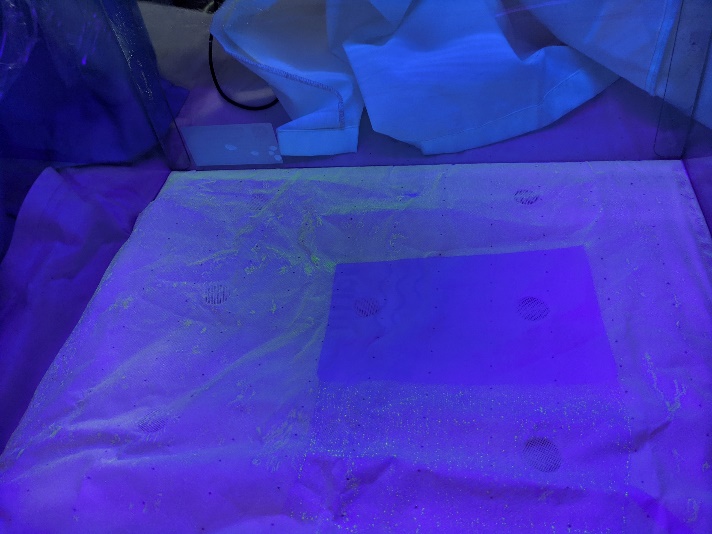
**

**
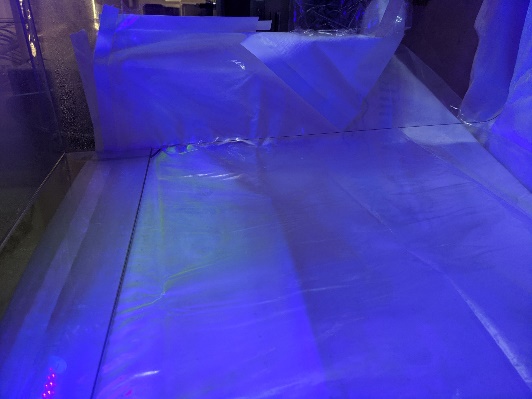
**

**S8 Figure. Images of fluorescent particle settling and escape visualization experiment**. Particles containing fluoresceine salt and illuminated via UV light are visible in yellow/green color. Nearly all visible particles are either sprayed on the wall directly on the path of the airbrush spray (a) or settled on the bottom of the enclosure (b). No visible signs of escape were visible. Blank space in (b) was where the nebulizer was located during testing.
