# Supplementary material for: Preventing spread of aerosolized infectious particles during medical procedures: A lab-based analysis of an inexpensive plastic enclosure
Source: PLoS One. 2022 Sep 22;17(9):e0273194. doi: 10.1371/journal.pone.0273194 (PMC9499281; doi:10.1371/journal.pone.0273194)
Supplement: Attachment — Submitted filename: COVID_PLOSOne_Reviewresponse2.pdf [file pone.0273194.s010.pdf]

The authors thank the Editor and reviewers for taking the time to further assess the quality of our manuscript. We understand the concerns conveyed by the Editor regarding taking too strong of a position on something that still warrants further testing by the medical community in a hospital setting and have revised the manuscript to either remove or rephrase these statements. These changes were largely made to the Abstract, Introduction, and Discussion sessions.

The changes to the manuscript are below. A few minor grammar edits and small deletions/additions are not shown here. If a passage includes text that was not deleted for context, the portion remaining with any additions are colored in blue. The deleted part is shown using ~~strikethrough~~. *New text is shown in italics.*

Abstract:

- "This enclosure is an inexpensive, easily implemented additional layer of protection that can be used to ~~reduce the risk of infectious aerosol transmission~~ *help contain infectious or otherwise potentially hazardous aerosol particles while enabling provider access into the enclosure*." ~~between patients and healthcare workers without greatly inhibiting provider access or satisfaction.~~

Introduction:

- "…this enclosure will still provide an effective further layer to *reduce the exposure to potentially hazardous aerosol*." ~~protection to healthcare workers.~~
- "This simple inexpensive enclosure with plastic barrier coverings can help to reduce the spread of viral aerosol particles and other hazardous aerosol." ~~in hospital and other health care settings for both the protection of health care providers, as well as their patients.~~

Discussion:

- This study investigates the use of an effective, inexpensive, *simple to use*, and easily sterilizable enclosure that utilizes accessible disposable plastic covers. Survey ~~evidence~~ responses from ~~by~~ medical professionals indicates that the enclosure can be used *satisfactorily* without hindering their work. *The large side openings with plastic covers enable easy access into the enclosure.* ~~It can be readily implemented to minimize the risk of airborne disease transmission to healthcare providers during tracheal operations.~~
- Depending on the amount of suction applied, a 5 to 20-minute waiting period following the last particle generation event is required to allow 90% of *the* particles to settle or be suctioned out before the enclosure ~~can be~~ *is* ~~safely~~ removed. ~~while avoiding exposing providers to elevated doses of potentially infectious aerosol.~~

- This statement at the end of the Discussion was deleted:
  "This enclosure provides further protection to health-care workers and patients from infectious aerosols. The enclosure is inexpensive, robust, easily manufactured on a large-scale, simple to disinfect between use, and can be used in virtually any hospital setting. Large openings draped in plastic provide enough space for healthcare workers to access the patient for procedures, without reducing the efficacy of particle entrapment. We recommend the use of this enclosure to capture potentially viral aerosols to reduce the spread of disease in a hospital setting."
