# Supplementary material for: Preventing spread of aerosolized infectious particles during medical procedures: A lab-based analysis of an inexpensive plastic enclosure
Source: PLoS One. 2022 Sep 22;17(9):e0273194. doi: 10.1371/journal.pone.0273194 (PMC9499281; doi:10.1371/journal.pone.0273194)
Supplement: Attachment — Submitted filename: Response to Reviewers.docx [file pone.0273194.s009.docx]

We thank the editor and the reviewers for their time in reviewing this manuscript and providing their feedback which has helped us to further improve the quality and presentation of our research. We have made a good-faith effort to adjust the manuscript and conduct additional experiments to address the main reviewer comments. Reviewer comments are in blue and our responses are below in black. Manuscript text changes are provided in quotes below each response. If context from surrounding text was deemed useful for understanding a revision of the manuscript text, *the new text is italicized*, unchanged text is not.

A new set of experiments was conducted to address comments by the reviewers examining how relative humidity changes in response to the nebulization of aerosol particles. Another attempt was made at measuring concentrations of aerosols produced by the aerosol generation method without an enclosure present. The new experimental results, or why the no enclosure experiment results were not used, are discussed with the relevant review comments.

The substantive revisions made to the manuscript sought to address reviewer comments as well as better place the study within the broader scientific context for understanding the performance of aerosol barrier enclosures such as studied here. The current FDA guidelines for these types of barriers still recommends their use with active suctioning (negative pressure) as the known benefits outweigh the risks (<https://www.fda.gov/medical-devices/letters-health-care-providers/protective-barrier-enclosures-without-negative-pressure-used-during-covid-19-pandemic-may-increase>). This is an emergency use authorization established using limited data available at the time of its publishing, and new designs, surveys, and experimental results will be used to inform future decisions. It is to that point directly that this study aims to provide better information regarding how the design choices for these enclosures affects their performance in containing aerosol inside and thus helping to reduce exposure of healthcare providers to potentially infectious or otherwise hazardous aerosol. While the first reviewer raises good concerns regarding the use of such barriers for medical purposes, these concerns are largely based on a quite incomplete understanding of how these barriers perform, and on earlier enclosure designs that certainly had significant flaws such as the lack of coverings or seals over the necessary openings in the enclosure. Our study responds to this need for better evaluation using proper aerosol science and technology approaches of how improved enclosure designs perform their intended purpose of containing aerosol produced inside them. This information is necessary so that health authorities such as the FDA can make better informed decisions and recommendations regarding how to best respond to the COVID-19 pandemic, and other health crises in the future.

# Reviewer 1:

In this manuscript the authors have created a ‘low-cost’ patient enclosure, generated aerosol inside the enclosure (with a solution of aqueous ammonium sulfate, azelaic acid and sucrose in deionized water) using a nebuliser to simulate breathing and a paint spraying airbrush to simulate cough. The authors used two condensing particle counters (CPC), a DustTrak aerosol monitor and an aerodynamic particle sizer (APS) to measure particles. Aerosol was measured inside and outside the enclosure to determine a ratio. One CPC was used within the enclosure, the Dustrak was generally used inside the enclosure and the APS generally used outside the enclosure. Only CPC values are reported to be shown in the paper.

A 30 second application of the airbrush was performed inside the enclosure. The authors found having uncovered apertures allowed 30% +/- 10% of the aerosol to escape. The application of a single layer of plastic over the apertures reduced the ratio of aerosol escaping and the addition of a second layer of plastic further reduced the ratio of aerosol that escaped. 2 minutes of the nebuliser within the enclosure also demonstrated occluding the hole reduced the amount of aerosol that escaped.

The authors tested the effect of suction at 15LPM and 30LPM on aerosol clearance and found suction decreased the time for aerosol concentrations to reduce to 90% of the peak. Fluorescein was nebulized to visually examine for areas of aerosol escape and deposition patterns within the enclosure.

We thank the reviewer for thoroughly reading and commenting on our manuscript and providing this detailed summary of our work. We focus on the CPC data as that is the most sensitive and reliable measurement of particle concentrations and was used to assess what fraction of aerosols escaped from inside the enclosure. The APS and Dustrak instruments measure the aerosol size distribution. This data was used to assess the size distribution of the aerosol generated inside the enclosure for our experiments.

The authors also conducted a survey of the enclosure to assess medical staff acceptability (n=39 respondents in 2 healthcare settings). It is unclear how this was performed as the manuscript references to medical staff “who performed tracheal intubation and extubation procedures” but the survey questions indicate this was simulated with mannequins.

We have clarified in the manuscript that the survey questions pertained to the initial use of the enclosure with mannequins by changing the title of survey results table to “Table 1: Survey of Medical Staff Performing Intubation/Extubation with Enclosure *on Mannequins* (n=39).” The survey was done based on use with mannequins since a research study using live patients is much more difficult to establish and have approved and there was a need to move quickly to address the pandemic. The staff surveyed are also experienced in working with live patients.

Text change also in supplemental:

# “Complete survey results of medical professionals that had used the enclosure for *simulated* intubation/extubation procedures *on mannequins* at 2 different hospitals”

Evidence quantifying the amount of aerosol generated from intubation and extubation in a real clinical environment has demonstrated these procedures does not generate aerosol. As such there is no evidence aerosol containment devices are required for these procedures [1,2].
The references cited by the authors pertaining to aerosol generation with intubation, extubation and suctioning do not support their statement. Simpson et al simulated aerosols generated by healthy volunteers coughing with a nebuliser placed in front of them [3]. Lindsley et al used coughing and breathing simulators to generate aerosol [4], neither of which reflect real clinical practice for intubation, extubation or suctioning.

As referenced by the authors, breathing, speaking and coughing does generate aerosol so the clinical applicability of this enclosure would require it be used at all times when a patient with a respiratory infection is conscious.

We reworded parts of the manuscript to account for the papers presented by the reviewer, including the removal of ‘aerosol generating procedure.’ We also clarified the role of the cited studies (3,4) were to inform the possible aerosol size distribution and how to replicate them. The intent was not use them to replicate the exact distribution during or after intubation, extubation, or suctioning.

We disagree with the framing presented by the reviewer that because these are not aerosol generating events then there is no need for such a device. COVID-19 is known to transmit from aerosols produced by passive breathing, in addition to aerosol produced by talking and coughing. Indeed, a major challenge in studying COVID transmission is that the number concentration of aerosol produced by breathing, talking, coughing, etc. is quite low compared to typical background aerosol concentrations and yet aerosol transmission is now understood to be a major transmission vector for COVID and many other infectious diseases.

Klompas, Baker, and Rhee lay out a framework that evaluates what qualifies as an aerosol-generating procedure [What is an Aerosol-Generating Procedure, *JAMA Surgery*, 2021]. They outline four key factors that in various parts or in whole can quantify a procedure that increases risks of infectious aerosol exposure to health providers: forced air through air passages, symptoms and severity of the disease, distance to the source, and duration near particle source. While we agree that the amount of aerosols directly generated by intubation and extubation alone would not quantify it as an aerosol-generating event such as a cough, the three other factors can make the risks faced by health providers similar. They are by necessity working with patients with severe cases, at a close distance, and for a non-trivial amount of time. The enclosure seeks to reduce the overall risk associated with those three other key factors. To remove confusion in the manuscript we do not refer to these as aerosol-generating events, but we maintain that there are still increased risks associated with these procedures.

We do not seek to provide medical advice here, only to document the performance of a simple enclosure that can be used to contain potentially infectious aerosol produced by a patient in situations where they cannot wear a mask for some time periods or medical operations. The FDA did issue an emergency use authorization for such enclosures as one of many measures that can be used to reduce transmission of SARS-CoV-2. As for non-operational times, we did not set out to replace the use of a medical mask and point out that they are still a viable option.

The updated Emergency Use Authorization regarding such enclosure devices from the US FDA still approves the use of actively ventilated (or negative pressure) enclosures. The EUA for passive enclosures was revoked due to concerns that this can create a risk to the healthcare provider but we note that this was based on the use of earlier enclosure designs without coverings as tested here. The FDA believes the benefits to using negative pressure enclosures outweighs the additional risks these may produce:

“If electing to use a protective barrier enclosure for additional protection during aerosolizing procedures by HCPs, FDA recommends the use of devices that incorporate negative pressure. FDA has authorized the use of several negative pressure barrier enclosures, which can be found on FDA’s Emergency Use Authorization website. Based on detailed review of data showing decreased HCP exposure to airborne particles, usability, and other safety and performance measures of negative pressure devices, the FDA continues to believe that the known and potential benefits for emergency use of these devices, when used as authorized, continue to outweigh the known and potential risks and do not present public health or safety concerns at this time. Although it is unknown whether negative pressure devices have the potential for similar complications as passive devices, at this time, the Agency does not have reasons, or evidences of any adverse events to believe that is the case. FDA is constantly monitoring for signals, and available literature related to authorized devices for mergency[sic] use.” (https://www.fda.gov/medical-devices/letters-health-care-providers/protective-barrier-enclosures-without-negative-pressure-used-during-covid-19-pandemic-may-increase)

Text changes in Introduction:

“Evidence suggests that current levels of personal protective equipment worn by providers are adequate to meet the needs of medical staff in preventing the spread of infectious aerosol particles, *particularly during extubation, intubation, and suctioning when new aerosol generation is not above background human emissions levels.* [38–40].

“While the medical procedures targeted in the design of the enclosure are not generally considered aerosol-generating, that does not preclude the risk of transmission during their operation, nor does the design intent limit the enclosure’s potential applicability to any procedure that requires an unmasked patient [39,40].”

Text changes in Methods:

“Generated particle concentrations were significantly higher than those observed by human coughing (Fig S3). This was done to simulate ‘worst-case’ scenarios and amplify the aerosol signal that escaped the enclosure, facilitating reliable measurements. *The over-abundance of smaller particles in the hundreds of nanometers diameter range was a result of the nebulizers used, but it was not seen as a limitation for this study. These particles have less inertia than larger particles and are better able to avoid impacting on obstacles and thus provide a more challenging test scenario of the enclosure’s ability to prevent particle escape. Generating aerosol that more accurately represents the total emissions and particle size produced during human coughing, or during intubation and extubation procedures, would have created conditions in the enclosure that were less likely to lead to particle escape.*”

Recent studies and a meta-analysis have demonstrated aerosol containment devices increase time to intubation, increase first time failure rate, are more likely to breach PPE and have potential to cause patient harm [5-8]. This is supported by reference 39 from the manuscript which states:

“The FDA is revoking the umbrella emergency use authorization (EUA) for passive protective barrier enclosures (those without negative pressure.) We have carefully reviewed and considered preliminary evidence showing that there is a potential for adverse events or complications when using these devices while treating patients who are known or suspected to have COVID-19.”[9]

A further statement by the Journal Anaesthesia referring to intubation boxes/enclosures states:

“these devices were produced outside the normal regulatory framework, and thus were never clinically tested or validated for effectiveness and safety. They were subsequently heavily promoted in the media and on social media. Yet despite this heavy promotion, no international guideline on personal protective equipment (PPE) has ever endorsed their use.” [10]

As such this aerosol containment device is neither required or validated and is likely to increase the risk of harm to both patients and healthcare workers.

We refer the reviewer to our initial comment at the top of our response regarding the need for better information and evaluations of such enclosure devices, especially of newer and likely improved device designs. The reviewers have raised important considerations, and a major objective of our study was to provide quality aerosol measurements to better evaluate the performance of such containment devices that can then inform or prohibit their use. We note that the revocation of the EUA was specific to passive enclosures without negative pressure/ventilation, while enclosures with negative ventilation are still recommended by the FDA’s EUA. We evaluated both types of enclosures here. We also point out that the revocation of the EUA for passive barriers was based largely on earlier simple enclosure designs without proper coverings over the large holes in the barrier, whereas here we find that the proper combination of such coverings can effectively contain the aerosol inside the enclosure. We have revised the introduction to clarify this as we discuss below.

We emphasize that the objective of our study is to evaluate the performance of a simple enclosure in containing aerosol produced from inside the enclosure, such as by a patient. We do not seek to make firm recommendations regarding its use in medical settings, even though this enclosure has been successfully used in a large hospital network. Our focus is on reporting the basic aerosol science aspects of how the enclosure performs, such that this data can help better inform the suitability of using such barriers should the need arise to contain potentially infectious or otherwise hazardous aerosol in a medical or other setting. As it is almost certain that we will experience other infectious disease outbreaks that are transmitted by aerosols, and because bioweapons can also be aerosolized, any new information regarding how to contain aerosol using readily available and simple materials that still permit access inside the enclosure is valuable and should be reported. We cannot foresee how this new knowledge might be applied in the future and argue that it is important to not prohibit disseminating such findings based on a specific intended use of the enclosure. We again emphasize that the FDA’s guidance regarding barrier was indeed based on a very incomplete understanding of the performance of such barriers, and how barrier design choices can influence the performance. This motivates the need to perform evaluations of such barriers as we have conducted here and to report these findings so they can better inform future recommendations from health agencies. We would ask the reviewer to consider the negative effect of not publishing these findings on our understanding of how these barriers perform which is needed to better address the same excellent concerns that the reviewer has raised.

We set out to design an enclosure to overcome some of the flaws associated with other similar enclosures by widening the handholes and increasing visibility. However, we don’t have the data to prove, nor would we claim, that the concerns brought up here by the reviewer are unfounded. It is up to each medical provider or agency to decide the best approach for their situation in accordance with their country’s regulatory limits. As such, all possible information regarding possible approaches should be made available to them to make the most informed decision. The performance of this enclosure in containing the aerosol generated inside it is far superior than earlier designs (such as with uncovered openings and no active ventilation) that became widely used early in the COVID pandemic but without a good understanding of their performance. The concerns raised regarding the use of such enclosures were derived largely from the use of these earlier device designs that do not directly relate to the use of this type of enclosure with coverings and also optionally negative pressure applied which was designed to address some of these major issues and then extensively evaluated using sensitive aerosol instrumentation.

The dynamics of the risk assessment and recommended precautions may also very in the future given a different infectious disease that presents different hazards, transmission and infection characteristics, and pathology. At the start of this current pandemic, we found even countries with highly funded and well supplied medical facilities were resorting to trash bags and other unconventional forms of PPE, for example. It is feasible that this will be the case again in the event of some future pandemic. Having a body of literature already available that describes possible non-traditional protection measures could prove more useful than having to create one in real-time. Were such information available before the onset of the pandemic our response taken could have been much different, more effective, saving many lives. However, we lacked a good basic understanding of how SARS-CoV-2 was transmitted, and once the aerosol transmission vector was identified and began to be accepted by the medical community, we lacked a good understanding of the most effective ways to reduce aerosol transmission and exposure. By reporting such new findings as we do here, we add to our body of knowledge that can be used in unforeseen ways for societal benefit in the future.

Text changes in Introduction:

“This enclosure design with coverings and the use of suction conforms to the revised FDA emergency use authorization released on August 21^st^, 2020 for such medical devices [39]. *The current FDA guideline prohibits the use of passive enclosures. Yet for enclosures with active suctioning (negative pressure), the FDA “believes that the known and potential benefits for emergency use of these devices, when used as authorized, continue to outweigh the known and potential risks and do not present public health or safety concerns at this time” [41]. This emergency use authorization is based on the understanding of the performance of the enclosure designs in wide use at the time of issurance. Studies such as this help provide a better understanding regarding how barrier design choices affect the performance of passive and active enclosure design for future consideration by regulatory bodies and healthcare providers*. While the use of active filtration was necessary for other similar enclosures without added coverings to reduce aerosol escaping the enclosure, we saw no evidence that active filtration decreased particle leakage from this enclosure with coverings and the corresponding funneling of particles towards the providers [24]. Therefore, in situations where active filtration is unavailable, such as in rural and resource-limited communities, and remote or mobile operations, this enclosure will still provide an effective further layer of protection to healthcare workers. *Active suctioning was found to greatly reduce the amount of time needed for aerosol loss via particle deposition to surfaces prior to removal of enclosure.* This simple inexpensive enclosure with plastic barrier coverings can help to reduce the spread of viral aerosol particles and other hazardous aerosol in hospital settings for both the protection of health care providers, as well as their patients.”

There is no costing data as to the low-cost element, no cleaning data as to the ability to clean/sterilise/disinfect and no data on how easy it is to construct despite. There is no patient acceptability data.

Thank you for pointing out this omission. We included pricing for consumables and approximate cost per use ($8-$10) in the supplemental. The cost of the enclosure was $350 per unit. This cost is variable based on the size of the order, manufacturer, and other factors.

Text changes in Supplemental:

“Suggested moving wrap can be obtained for less than $40 (USD) to cover 41 containers. 3M Steri-Drapes can be found for $100 per 40, and $120 per 12 upper body blankets, with 0.5 used per enclosure use. This puts costs of disposables at less than $10 per use of the enclosure. The build price per unit was $350, however, that price could vary for different users.”

There has been no attempt to “simulate an airway procedure”. By inserting an operator’s hands into the box and removing them does not reflect real world practice of airway manipulation.

We agree that our experimental setup did not replicate a medical procedure. To conduct such experiments on human subjects would of course require considerably more time, effort, and approval. We reworded the methods section to clarify this as well as further describe the hands test as a full minute of insertion, movement inside the enclosure, and removal of the hands from the enclosure. Moderate jolting and shaking of the container was also conducted. The insertion and removal portion of the hands were the times most likely to increase particle escape from the enclosure, and we do not expect that movements specific to the medical procedure under realistic conditions would substantially increase particle escape from the enclosure.

Text changes in Methods:

“In some experiments, an investigator’s hands wearing nitrile gloves were inserted through the hand holes cut in the front covering, *moved within the enclosure for one minute*, and then withdrawn to *mimic motions and stresses placed on the enclosure during field use*.”

The manuscript states all data are reported “in particle number per cm-3” (line 172) however there are no particle concentrations reported throughout the manuscript or supplementary material.

We clarified to indicate that the data reported is a ratio of the particle concentrations measured inside to outside the enclosure and not the particle concentrations themselves. The aerosol instruments used measure aerosol number concentrations per volume of air (particle number per cm^3^).

Text changes in Methods:

“…all data reported in this study were collected in particle number per cm^3^ *and then normalized by dividing maximum external concentration by maximum internal concentration*.”

There is no data on the background aerosol concentrations or where the study was performed.

Particle background concentrations varied between days but averaged between 500-2000 cm^-3^. These are typical levels for indoor environments. All runs sampled background air for at least 1 minute prior to the particle generation event. This information has been included in the supplemental. Further descriptions of the lab environment where the experiments were performed – an air quality lab at Carnegie Mellon University in Pittsburgh, PA – were also included.

Text changes in Supplemental:

“Background particle concentrations were 500-2000 per cm^3^ depending on day. Peak internal concentrations varied by nebulizer and enclosure setup but ranged from 5000-100000 per cm^3^. The upper range likely experienced under counting issues due to overlapping particle “coincidence” detection events in the CPCs for concentrations > 10^5^ cm^3^.

The lab testing was conducted in the Air Quality Laboratory at Carnegie Mellon University in Pittsburgh, PA during the spring and summer months. Environmental HVAC controls kept general lab RH and temperature fairly consistent throughout experimentation.”

It is unclear how many experiments were performed. Throughout the manuscript the authors report "in some experiments" or "in a few experiments".

We added this important information to the methods; each experiment type included 4-5 individual runs.

Text changes in Methods:

“All experimental data reported were the result of four or five replicates.”

The sampling locations for each experiment are unclear, sampling was performed at a number of locations but it is not clear when or for which experiment. The authors report “near the hand holes, near the side hole, or along the base at the back” and “numerous measurements were taken around the entire enclosure”.

To clarify this, we included a figure in the supplement that indicates the exact location of external sampling locations in reference to the enclosure in supplemental:

“Figure S4. External aerosol sampling locations near the enclosure marked by a marker. Blue circle indicates sampling location when measuring in the no covers or one cover with hand slits configuration. The red crosses indicate locations used for dual cover configuration measurements. The green square indicates other sampling locations tested near the side hole and further away from the enclosure. All internal sampling lines and vacuum hose were inserted through the side port. Nebulizers were placed at the center of the enclosure and pointed 70-90º above horizontal table level between 12-17 cm above the bottom surface, depending on which type of nebulizer.”

The use of visible fluorescein is a poor surrogate marker for aerosol deposition due to the differences in the size range of visible fluorescein compared to aerosol.

We expanded on this section to discuss that the fluorescein provides a good surrogate for areas of high particle deposition that would require more attention during cleaning. We also added that the size range of the particles seen observable with the fluorescein is about 50 µm, too big to be the particles of most concern. However, smaller size ranges were known to deposit on the sides of the enclosure. This was tested by moving a finger along areas without visible deposition. The finger acted to coagulate the particles and increasing their fluorescein residue such that we could assess if particle deposition had occurred on that surface. While fluorescein has its limitations, as do all methods, it is a useful way to gain insights into where particle deposition occurs in the enclosure. It is commonly used to assess particle deposition when designing aerosol instruments, for example. Other approaches that are more sensitive to smaller aerosol are much more difficult to apply here as they would require optical microscopy examination. The text was modified as follows:

Text changes in Results:

“To determine whether particles were depositing, and where, experiments with fluorescent particles were conducted. Results indicated no particle leakage in the visible particle size range (approx. 50 µm) from the enclosure without suction (S5 Fig).”

Although acknowledged by the authors, Figure S3 demonstrates the simulated aerosol in this study is not representative of that generated by a physiological cough (being 1-2 orders of magnitude greater).

Creating a completely accurate size distribution of particles in the enclosure required either human subjects or custom engineering. We instead focused on nebulization devices that would produce particles up to the size created by humans as well as a sufficient number concentration of particles that enables the measurements required to assess the enclosure’s performance. The fact that the generated particle distribution is higher in concentration but smaller in size than that produced by humans provides a more challenging aerosol condition to assess the ability of the enclosure to contain the aerosol. Smaller aerosols corresponding to the generated aerosol size range have longer airborne lifetimes (slower rates of gravitational settling as well as Brownian diffusion) than larger aerosols that better represent human-produced aerosol. Thus, the smaller nature of the generated aerosol is more prone to escaping the enclosure than the larger aerosol is. When evaluating aerosol containment or filtration the most penetrating aerosol size is typically focused on (~0.3 µm in diameter for filtration such as using a mask). We clarified this in the text as follows:

Text changes in Methods:

“The over-abundance of smaller particles in the hundreds of nanometers was a result of the nebulizers present, but it was not seen as a limitation for this study. These particles are better able to navigate obstacles and test the enclosure’s ability to prevent their escape. Closer accuracy of the total emissions and particle size during human coughing, or during intubation and extubation procedures, would have created conditions in the enclosure that were less likely to cause particle escape.”

There is a mix of imperial and metric units throughout the manuscript and figures.

Thank you for pointing these out. The imperial units in Figure 1 and length description of covering plastic have been converted to metric.

Data analysis has been performed with a mix of parametric and non-parametric tests without justification.

This is a good point. Part of this is due to the nature of the data from inside the enclosure versus that outside the enclosure. The behavior of the particles within the confined space of a fully covered enclosure was significantly more consistent than that of an uncovered enclosure or outside the enclosure. A similar dynamic was seen with the distance measurements external the enclosure. We did consult with a statistician for all testing parameters and feel that the statistics are robust enough to reliably assess the measured data and address our stated objectives in evaluating the enclosure performance.

Text changes in Supplemental:

“Of note is the difference in use between parametric and nonparametric tests. The wide difference in consistency between data collected inside the enclosure, close to the enclosure, and far from the enclosure made consistent use of exact statistical testing parameters difficult. In general, data collected within the enclosure and near the enclosure was widely reproducible and generally followed assumptions associated with parametric testing. Dilution and the rapid dispersion of aerosol once outside the enclosure are the root drivers behind the differences in the degree of variability of the different aerosol measurement locations.”

# Reviewer 2:

This manuscript (PONE-D-22-01962) reports measurements of aerosol escape from a plastic enclosure designed to mitigate risk of exposure to infectious respiratory aerosols and droplets generated during clinical procedures like intubation and extubation. The plastic enclosure consists of several openings to accommodate the patient, the provider, and relevant medical instrumentation. In some cases, these enclosures were open, whereas in others they were covered using a range of plastic coverings. Aerosol was generated inside the enclosure using different medical nebulizers and was measured in the ~0.1-10 μm size range using a suite of aerosol measurement equipment that included condensation particle counters, scanning mobility particle sizers, and aerodynamic particle sizers. Aerosol escape, quantified by the ratio of maximum number concentration outside and within the enclosure, and the decay rate of aerosol generated within the enclosure were used to infer the effectiveness of the apparatus for mitigating exposure to potentially infectious aerosols.

This manuscript reports on a type of apparatus that has had significant use during the COVID-19 pandemic. The measurements made are largely appropriate, and this work is timely. The work is within the scope of PLoS One and will be publishable once the comments below are satisfactorily addressed. Given the subject matter of this manuscript, it is worth noting that this reviewer’s expertise is in aerosols and aerosol generating procedures; this reviewer has no clinical expertise.

We thank the reviewer for taking the time to read and comment on our work and recognize the timeliness of our research in regards to the continuing COVID-19 pandemic.

Lines 31-32 (“should be left on the patient for 15-20 min”): This seems like a very long time and potentially impractical if this apparatus were widely used. More broadly, how do these decay rates compare to those typical of the clinical environment in which these procedures usually occur? In their revised manuscript, the authors should comment more explicitly on the practicality of the apparatus given the results of their study.

It is a long period of time in a hospital setting. We updated our recommendation to use active suctioning wherever possible to reduce the amount of time the device must be kept over the patient, but we want to cover the widest possible use scenarios. This is expected to include places without active suctioning infrastructure. In times of limited operating room availability, the enclosure is designed to fit on a standard operating bed, and the patient could still be transported to other rooms with the enclosure in place to reduce the need to remove the enclosure before sufficient time for particle settling has occurred. While the aerosol would also dissipate as it mixes with the room air, the operating rooms where these tracheal operations are conducted are often small in size, and this dilution by entrainment would still expose healthcare providers to potentially infectious aerosol, which the use of active ventilation prevents.

Text changes in Discussion:

“The main benefit of adding active suction to the enclosure is to reduce suspended particle lifetimes in the enclosure, *and it is suggested that suction is used wherever feasible*. *The FDA has provided an emergency use authorization for barriers such as these only with negative pressure applied and rescinded its earlier approval of passive barriers without active filtration.”*

Line 206 (and following discussion of Fig. 3): For these measurements, presumably the concentration inside the apparatus was relatively homogeneous due to the enclosure, but external to the apparatus there would be substantial dispersion, which would result in very rapid decreases in number concentration with distance from the opening. The authors should discuss this complication and how it was considered in the experimental protocol and data analysis. Is it possible that the number concentrations measured at 8 cm from the box are simply an artefact of the sampling position and may significantly underestimate aerosol leakage? The authors’ revised manuscript should enhance their discussion of this measurement, as it is a critical aspect of the manuscript and requires more careful discussion.

This is a good point though it would apply to any situation or use case scenario as aerosols quickly experience dispersion as they mix with the surrounding air. However, even low concentrations of virus-containing aerosol pose a serious infection risk and thus we used very sensitive particle counting instruments that can better assess how much aerosol is escaping from the enclosure. The most relevant comparison cases are the experiments performed with and without covers over the openings in the enclosure. When measuring which location to be the primary sampling sites, we also varied distances. We did not observe a significant difference in the particle concentration ratio for distances less than 8 cm. The aerosol flow drawn from the CPCs is enough to be the strongest air flow source over short distances such as this resulting in decent particle sampling. The flow rate is small though (0.3 lpm), limiting the overall air volume that the CPC samples from. This would account for much of the observed decrease in particle concentrations as the distance increased past 8 cm away from the enclosure. We have added a paragraph to the discussion section detailing further how this distance could affect particle measurements of particle leakage.

Text changes in Discussion (entire quote is new addition):

“The size of the enclosure and limited aerosol measurement instrumentation made monitoring all possible leak points impossible. However, care was taken to monitor places most likely to yield particle leakage during normal use (Fig. S3). These areas were where two materials met but could not be sealed with tape due to the necessity of access. At the primary sampling range of 8 cm, the air intake of the instruments would draw in many particles, particularly for the smaller particles which are more likely to escape. The strength of this draw diminishes rapidly with increasing distances and can help account for the decreasing measured concentrations at greater distances as particles had more volume to rapidly disperse and avoid detection.”

Lines 213-214 (“trapping efficiency”): Is “trapping efficiency” the most appropriate word? The incorporation of suction just means that aerosol preferentially will follow the gas flow to the suction device, reducing the amount that escapes through the other openings.

We agree. We reworded this sentence to:

“Adding 15 LPM suction to the Steri-Drape plus furniture wrap configuration *reduced external aerosol concentrations by* 98 ± 3% *over internal concentrations*.”

Lines 238-239: The authors should consider whether it might be appropriate to do a control experiment to see how number concentration decays with distance from the aerosol generation in the absence of an enclosure. Ultimately, the authors are reporting a concentration at some distance, but that concentration necessarily will naturally reduce simply due to dispersion. While aerosol might be homogenously dispersed within the apparatus, that will not be the case in the surrounding room. The authors need to address this challenge more directly in their revised manuscript.

In response to this suggestion, we did attempt a no box set of experiments as an additional control to compare to. However, there is constant air flow through instrument/tubing exhaust lines in the lab being used. This suction was powerful enough to draw aerosol particles generated without the enclosure in a manner not represented of natural diffusion and mixing into still air. This will be the case for any indoor environment with active ventilation, such as a hospital setting. The enclosure without covers was sufficient to disrupt this ambient air flow and allow the particles to behave in a more natural unforced manner. Our sampling lines were always placed ‘downwind’ of potential leak sites relative to the prevailing air flow in the laboratory. The measurements as a function of distance away from the enclosure (made as close as 7.6 cm) provide a good assessment of the number fraction that escapes from inside the enclosure, and how quickly these aerosols become entrained into the background air with its much lower background aerosol concentration < 2000 cm^3^. The most relevant comparison are the experiments with and without covers placed over the enclosure as these largely capture the effect that covers placed over the openings have on containing the aerosol inside the enclosure. It is already known from prior studies that such openings if left uncovered tend to focus the aerosol and direct it through these available openings into the surroundings.

Text changes in Supplemental (entire quote is new addition):

“A control experiment with no enclosure was not possible in the available lab space. Permanent exhaust lines/snorkels in the lab ensure a constant directional air flow in the lab. Nebulized particles completely open to room air followed this flow. A similar situation would be present in any indoor environment with active ventilation and/or active personnel, such as a hospital setting. The enclosure without coverings configuration was enough to disrupt this ambient air flow and sampling lines were placed ‘downwind’ of any opening relative to the prevailing air flow in the laboratory. The measurements as a function of distance away from the enclosure (made as close as 7.6 cm) provide a good assessment of the number fraction that escapes from inside the enclosure, and how quickly these aerosols become entrained into the background air with its much lower background aerosol concentration < 2000 cm^3^.”

Lines 248-249: Would it make more sense to fit an exponential decay and report the time constant (similar to estimating an air change rate)? Ultimately, this appears to be an appropriate parameter to describe the decay of the aerosol concentration within the apparatus.

The use of the half-life that we determined provides very similar quantitative assessment as the exponential decay rate. We feel that it has a broader understanding across varying scientific and medical fields and is ultimately more useful as a result.

Line 268 (“visible size range”): To what particle sizes does “visible size range” roughly correspond?

We have updated this in the manuscript to reflect approximately 50 µm in diameter. We are confident that this does capture a smaller size range as we are able to run a finger over areas with no visible deposition and coagulate the residual into something visible. We are not certain how small this size range goes. The material of the enclosure does appear prone to build up electrostatic charge, so it is feasible that many of the smaller particles experience significant loss as a result. However, capture of particles below 100 nm is not useful due to the virus itself being that size and human-generated aerosol is not composed of bare virus particles.

Text changes in results (in italics):

“To determine whether particles were depositing, and where, experiments with fluorescent particles were conducted. Results indicated no particle leakage in the visible size range *(approx. 50 µm)* from the enclosure without suction (Fig. S5). Most particle deposition was located on the bottom of the enclosure or directly in front of the spray nozzle. A fine film, noticeable when touched, was formed on the sides where suspended particles deposited onto the walls. This film was not observable prior to being agglomerated together through touch, indicating that *some* particles smaller than the visible size range are also being trapped and deposited, *likely through wall deposition and electrostatic attraction to the plastic walls of the enclosure.”*

Lines 340-342: The statements here overreach, as the outcome will depend critically on, for instance, infective dose. If the infective dose is very low, then the enclosure may not actually reduce chance of infection. However, it will reduce total exposure to potentially infective aerosol.

Thank you for pointing that out. We have adjusted the line to fit within scope accordingly.

Text changes in Discussion:

“The lack of corresponding increase in exterior particle concentrations suggest that the total exposure to potentially infectious aerosol particles providers are subjected to will be minimal.”

Figures S1 and S2: Are the size distributions in these two figures self-consistent? On first glance, the SMPS and APS size distributions look to be very different in the limited size range where there is overlap. However, this could be due to the very different number concentrations. The authors should compare these size distributions in more detail in their revised manuscript.

Yes, we have a known undercounting issue with this APS instrument below 1 µm (due to reduced scattering laser power). The OPC, as well as the manufacturer’s calibration assure us that sizes above 1 µm are counted accurately. As such, we put more stock into the SMPS measurements in the overlap size region. We have added that information to the supplemental.

Text changes in Supplemental:

“This specific APS is known to undercount particles below 1 micron. As such, when comparing concentrations in the region in which the SMPS and the APS overlap, the SMPS should be considered the more accurate measure.”

More generally, and related to size distributions, how did relative humidity in the chamber vary with time? If relative humidity is increasing with increasing nebulization time, the size distribution (though not the number concentration) may change substantially. In their revised manuscript, the authors should discuss in more detail the size distributions and stability of these distributions over time.

We conducted experiments to track relative humidity over time and have added the results to the methods section of the main manuscript. The trials indicate an increase in RH as a result of the nebulizer’s operation resulted in a 5 ± 2% (absolute) increase in RH before slowly going back towards background RH over the course of the 20-minute trial period (Omron with 3 minutes of nebulization. This is the longest nebulization period used in our study). This relatively small change in RH would have an insignificant impact on particle behavior. More generally, high RH should decrease particle evaporation rate and increase gravitational settling rate compared to a lower RH. Unfortunately, we do not have RH data going back to the main body of experiments, other than that they were at comfortable room ranges (around 30-70%). Given the short half-lives of particles inside the enclosure under active suctioning, and the very small fraction of particles that escape when two layers of covering are used, such changes in particle size and physics induced by changes in RH will not have significant effects under these minutes-long timescales.

Text changes in Methods:

“The longest period of particle generation, three minutes, was determined to increase relative humidity in the enclosure by 5 ± 2%. It is unlikely that this increase in relative humidity would have a significant impact on particle behavior over the minutes timescale relevant to the lifetime of particles in the enclosure when active suction is applied. Overall relative humidity varied by day but was within a normal “comfortable” range (~30–70%) for climate-controlled indoor environments.”

Text changes in Discussion:

“Particles remain larger for longer at higher relative humidity. This would increase the fraction of particles lost to gravitational settling, though this study did not investigate the extent of this impact [49].”

This manuscript is motivated by a desire to reduce clinician exposure during aerosol generating procedures. However, there is minimal discussion about advances made in quantifying aerosol generation during procedures like intubation (for instance, the series of papers from Brown, Shrimpton, and Pickering, e.g. <https://doi.org/10.1111/anae.15292>, <https://doi.org/10.1111/anae.15599>). The authors in their revision should place their work in the broader context of our current understanding of aerosol generating procedures.

Thank you for this suggestion. These recent papers have been added to the reference list, and some discussion about them added in the introduction and discussion sections. As a result, we have reframed these away from being ‘aerosol generating procedures.’ Despite the findings of these papers that demonstrate that these procedures do not generate excess amounts of particles, we still feel it is prudent to keep the use of such devices an option depending on each provider’s situation. COVID is known to transmit even at aerosol concentrations produced by normal breathing and talking, and these would also not be considered aerosol-generating procedures. Given the highly infectious nature of some SARS-CoV-2 variants, even these low aerosol concentrations represent a real exposure and infection risk given our current understanding that aerosols are the primary transmission vector for COVID.

Text changes in Introduction:

“Evidence suggests that current levels of personal protective equipment worn by providers are adequate to meet the needs of medical staff in preventing the spread of infectious aerosol particles, *particularly during extubation, intubation, and suctioning when new aerosol generation is not above background human emissions. [38–40].*”

“While the procedures targeted in the design of the enclosure are not considered aerosol-generating, that does not preclude the risk of transmission during their operation, nor does the design intent limit the enclosure’s potential applicability to any procedure that requires an unmasked patient [39,40].”

“This enclosure design with coverings and the use of suction conforms to the revised FDA emergency use authorization released on August 21^st^, 2020 for such medical devices [39]. *The current FDA guideline prohibits the use of passive enclosures. Yet for enclosures with active suctioning (negative pressure), the FDA “believes that the known and potential benefits for emergency use of these devices, when used as authorized, continue to outweigh the known and potential risks and do not present public health or safety concerns at this time” [41]. This emergency use authorization is based on the understanding of the performance of the enclosure designs in wide use at the time of publishing. Studies such as this help provide a better understanding regarding how barrier design choices affect the performance of passive and active enclosure design for future consideration by regulatory bodies and healthcare providers*.”

# Reviewer 3:

The introduction is well written and adequately describes the state of the science.

Thank you for the positive comment on our introduction.

Line 147. The size distribution of aerosol/droplets produced by a cough are commonly reported to be much larger than those used in the study (e.g. <https://royalsocietypublishing.org/doi/10.1098/rsif.2013.0560>). Why was this very small size region used?

This is a good point. In the practical sense, we did not have a nebulizer that would produce aerosols on the larger size of the cough range. However, we did not feel that this was a particular limitation to the study as such large particles have high inertia and are readily impacted on the surfaces of the enclosure either from expulsion or gravitational deposition. The aerosols large enough to contain a viral load but small enough to avoid quick gravitational settling (ca. 200 nm to 2000 nm) is what we wanted to make sure was most present in the enclosure for our tests. High amounts of aerosol in this size range is what we considered worst-case scenario for this enclosure as aerosols of this size have longer lifetimes and are more prone to escaping out of the enclosure.

Text changes in Methods:

“The over-abundance of smaller particles in the hundreds of nanometers diameter range was a result of the nebulizers used, but it was not seen as a limitation for this study. These particles have less inertia than larger particles and are better able to avoid impacting on obstacles and thus provide a more challenging test scenario of the enclosure’s ability to prevent particle escape. Generating aerosol that more accurately represents the total emissions and particle size produced during human coughing, or during intubation and extubation procedures, would have created conditions in the enclosure that were less likely to lead to particle escape.”

Line 137 The trajectory of an aerosol size is highly dependent on size, which itself is a by-product of both the starting solute composition and relative humidity. Both of these parameters are not reported in the main body of the manuscript.

We did not measure RH at the time of main data collection, but it was in the comfortable room range (30-70%). Evaporation can certainly change the aerosol’s size though this alone will not alter its initial trajectory. Aerosol size will affect other important aerosol physics such as its momentum, rate of gravitational settling, and Brownian diffusion (only relevant for very small sizes < 100 nm). However, all these effects also occur with human-generated aerosol that will enter indoor environments with wide ranges of RH as well as air exchange rates. These are certainly important effects but as the RH experienced by the aerosol in clinical settings will be quite variable it would require a dedicated study to properly assess this. Size-induced changes in particle physics such as their gravitational settling velocity are not that significant when compared to the minutes half-life of the aerosol inside the enclosure when two coverings and active suction are applied. As discussed above, because the generated aerosols are smaller than most human-produced aerosol, they will already have longer lifetimes and lower rates of interval loss and provide a more challenging test of the enclosure’s ability to contain the aerosol until it is lost by deposition or suction. The effects relevant to our experiments and aerosol used are captured by the aerosol size distribution measurements we obtained of the aerosol generated inside the enclosure. The particle composition was added from the supplemental to the main text, as well as information regarding the RH increase produced by the nebulizer.

Text changes in Methods:

“Particles were generated from a was 1 mg/mL ammonium sulfate aqueous solution.”

“The longest period of particle generation, three minutes, was determined to increase relative humidity in the enclosure by 5 ± 2%. It is unlikely that this increase in relative humidity would have a significant impact on particle behavior over the minutes timescale relevant to the lifetime of particles in the enclosure when active suction is applied. Overall relative humidity varied by day but was within a normal “comfortable” range (~30–70%) for climate-controlled indoor environments.”

Line 163 Using fluorescein to visualize the areas wherein the aerosol escapes and is deposited is problematic for a number of reasons. Only the largest droplets will settle out of the air and deposit on the surface. Additionally, the level of fluorescence will be proportional to the size of the droplets themselves (e.g. larger droplets will fluoresce more). Collectively, this measurement will provide only a limited understanding of the spread of only the largest aerosol droplets. While this may useful for fomite transmission, it tells very little about the aerosol that may be inhaled.

We agree with this observation and have adjusted the manuscript to make clearer the conclusions that can be drawn from this data. We expanded on this section to discuss that the fluorescein provides a good surrogate for areas of high particle deposition that would require more attention during cleaning. We also added that the size range of the particles seen directly with the fluorescein is about 50 µm, too big to be the particles of most concern. However, smaller size ranges were known to deposit on the sides of the enclosure. This was tested by moving a finger along areas without visible deposition. The finger acted to coagulate the particles and increasing their residue such that we could assess if particle deposition had occurred on that surface. While fluorescein has its limitations, as do all methods, it is a useful way to gain insights into where particle deposition occurs in the enclosure. This provides additional understanding regarding the performance of the enclosure. We rely primarily on the CPC measurements of aerosol number concentrations to evaluate how well the enclosure contains the generated aerosol inside. Fluorescein is commonly used to assess particle deposition when designing aerosol instruments, for example. Other approaches that are more sensitive to smaller aerosol are much more difficult to apply here as they would require optical microscopy examination. The text was modified as follows:

Text changes in

*“To determine whether particles were depositing inside or around the enclosure’s openings, and where, and where, experiments with fluorescent particles were conducted. Results indicated no particle leakage in the visible size range (approx. 50 µm) from the enclosure without suction as verified via visual inspection with a blacklight (S5 Fig)*. Most particle deposition was located on the bottom of the enclosure or directly in front of the spray nozzle. A fine film, noticeable when touched, was formed on the sides where suspended particles deposited onto the walls. This film was not observable prior to being agglomerated together through *wiping*, indicating that some particles smaller than the visible size range are also being trapped and deposited, *likely through wall deposition and electrostatic attraction to the plastic walls of the enclosure*

Line 172 Instruments were used to gather size information, while only the CPC data was provided in the main text. Surely more useful information is within the size dependant data. The size dependent data should be in the main text.

This is a good point that deserves further explanation, and we have added it to the supplemental as a result. There are several reasons why we focused on the CPC data. The CPC is the most sensitive and reliable measurement of particle number concentrations, over a wide size range. Since Covid transmission is largely driven by aerosol number (and not mass or surface area) this metric was the most suitable to evaluate the performance of the enclosure. Our specific APS instrument is known to undercount below 1 µm. This resulted in the OPC and APS having poor data overlap for comparison purposes. The integration time on the SMPS was too slow to capture the sudden changes in particle concentrations we might measure such as when taking hands in and out of the enclosure. The CPCs were the only instruments with a fast response time and good overlap between internal and external measurements. While this means we are putting a disproportionate amount of weight on the smaller, more numerous particles (even ones too small to contain a virus itself), we feel it provides a more robust overall evaluation of particle leakage.

Text changes in supplemental:

“This specific APS instrument is known to undercount particles below 1 micron, based on a recent manufacturer calibration (due to a low scattering laser power). As such, when comparing concentrations in the region in which the SMPS and the APS overlap, the SMPS should be considered the more accurate measure.”

“Thus, *considering the SMPS’s long integration time, the APS’s known undercounting of submicron particles, the OPC’s unique size range and large bins (PM_10_, PM_2.5_, PM_1_ and total)*, we defaulted to the CPC measurements for all figures and data shown in paper, *apart from S1 and S2*.

Line 177 Why wasn’t any statistical relevance calculated to demonstrated significance?
*Line 177 included for context: “Tubing connected to the CPC sampling ports was placed in areas deemed most likely to leak during data collection. These areas included near the hand holes, near the side hole, or along the base at the back.”*

All other areas of the enclosure came to a complete seal that would not likely leak particles. The enclosure was placed on padded fabric to simulate the surface of a medical bed. The enclosure was heavy enough to recess this fabric and provide a good tight seal on the three unopened sides. The edges of the wraps we used layered across the sides of the enclosure by 4 or more centimeters and were sealed with tape. The areas we selected to show were the only areas where possible gaps could reasonably be present. In the event of widespread use of this enclosure, it is possible that different approaches would be taken that would result in new routes of aerosol escape. As a result, we have added a section stressing the importance of proper sealing of the enclosure to minimize leakage.

Text changes to Supplemental:

“The enclosure was placed on top of a thick layer of fabric to act as a surrogate for a hospital bed.”

Text changes to Discussion:

“Proper sealing of the enclosure is critical to obtain high levels of particle retention within the enclosure. One advantage of the 50.8 cm plastic sealant is that it provides ample coverage around the front of the enclosure to minimize any chance of particle leakage through the edges. The users should take care to tuck in or seal with tape any other areas where particle leakage may occur particularly around the patient’s body or through the side port. Extra-long and heavy material on the back opening allow this to be achieved more effectively.”

Figure 1 A detailed drawing of where the aerosols are being sampled should be provided, as well as the location of the nebulizer used to produce the aerosol

This is a great observation, and a new figure has been included in the supplemental showing these locations.

“Figure S4. External aerosol sampling locations near the enclosure indicated by a marker. Blue circle indicates sampling location when measuring in the no covers or one cover with hand slits configuration. The red crosses indicate locations used for dual cover configuration measurements. The green square indicates other sampling locations tested near the side hole and further away from the enclosure. All internal sampling lines and vacuum hose were inserted through the side port. Nebulizers were placed at the center of the enclosure and pointed 70-90º above horizontal table level between 12-17 cm above the bottom surface, depending on which type of nebulizer.”

Figure 2 If no covers are present, the concentration immediately outside of the cover, should be the same as inside the chamber. This speaks to the point I’ve raised above. Unless the reader knows where the samplers are, it’s difficult to interpret this data. Moreover, “No Covers” is not an adequate control to measure the utility of the box. Rather, no box present at all should be considered.

In response to this suggestion we did attempt a no box set of experiments as an additional control to compare to. However, there is constant air flow through instrument/tubing exhaust lines in the lab being used. This suction was powerful enough to draw aerosol particles generated without the enclosure away in a manner not represented of natural diffusion and mixing into still air. This will be the case for any indoor environment with active ventilation, such as a hospital setting. The enclosure without covers was sufficient to disrupt this ambient air flow and allow the particles to behave in a more natural unforced manner. Our sampling lines were always placed ‘downwind’ of potential leak sites relative to the prevailing air flow in the laboratory. The measurements as a function of distance away from the enclosure (made as close as 7.6 cm) provide a good assessment of the number fraction that escapes from inside the enclosure, and how quickly these aerosols become entrained into the background air with its much lower background aerosol concentration < 2000 cm^3^.

Text changes to Supplemental:

“A control experiment with no enclosure was not possible in the available lab space. Permanent exhaust lines/snorkels in the lab ensure a constant directional air flow in the lab. Nebulized particles completely open to room air followed this flow. A similar situation would be present in any indoor environment with active ventilation, such as a hospital setting. The enclosure without coverings configuration was enough to disrupt this ambient air flow and sampling lines were placed ‘downwind’ of any opening relative to the prevailing air flow in the laboratory. The measurements as a function of distance away from the enclosure (made as close as 7.6 cm) provide a good assessment of the number fraction that escapes from inside the enclosure, and how quickly these aerosols become entrained into the background air with its much lower background aerosol concentration < 2000 cm^3^.”

Figure 3 What is the elevation that the samples are taken?

We are a little unclear exactly which elevation is being asked for here. Most sampling reported in experiments were taken at halfway up the enclosure from the base (roughly 32 cm), except for the back corners at the patient entrance. These were collected about 1-2 cm above table level. The nebulizer outputs were 12 cm or 17 cm above table level. To mimic a medical bed, the enclosure was placed on thick fabric that recessed under the enclosure providing a solid seal that was not a source of particle leak on the three sides of the enclosure that met the table. The elevation above sea level for the lab is approximately 233 m.

Text changes to Methods:

“Both nebulizers emitted particles 12-17 cm above surface level.”

Text changes in Supplemental:

“Sampling lines were located on the level of enclosure areas being measured: approximately 32 cm above table level for front and side ports, 1-2 cm above table level for patient entrance.”

Line 317 How do the authors know that 90% of sub micron droplets will deposit in 15 minutes. Are they not simply reporting the loss of aerosol detection? If that is the case, where is the aerosol going? My concern is that if this box is used to limit the exposure of aerosol, the authors should have a clear understanding what is happening to all of the nebulized sample.
In order for something to be defined as disinfected, around 99.999% of the infectious species needs to be inactivated. In this study, no where near that level of aerosol removal has been demonstrated. The authors need to make this limitation clear to the reader, and provide adequate context in the discussion.

This line has been reworded to accommodate the uncertainty pointed out here. While it is possible for us to identify all sources of major particle loss in these experiments, we cannot quantify them all. The one most easily quantified is particle deposition: 5 µm will cover the whole internal distance of the container in 14 minutes. All particle concentrations between 3-5 µm will be significantly reduced by gravitational deposition alone in the suggested window of 15-20 minutes (a 3 µm particle takes 38 minutes for complete deposition from the very top to the bottom of the enclosure, and 4 µm requires 21.5 minutes). The plastic enclosure exhibits strong electrostatic characteristics, and this electrostatic behavior is continually renewed by the hands-on nature of the procedure. This likely captures significant particle amounts in the smaller, sub-300 nm size range. The exceptionally high particle concentrations seen here would result in some degree of particle coagulation. The instrumentation itself cycled ~5% of the internal volume of the enclosure in this timeframe. The balance is likely lost to slow leakage. Internal aerosol measurements using the CPC confirm that total loss rate is significant, while external CPC measurements confirm that particle escape is not significant enough to increase aerosol concentrations above background levels even when measured close to the enclosure. When active suction is applied this significantly reduces the particle concentrations inside the enclosure and the time to achieve this reduction as most are now lost to the vacuum flow. Indeed, we recommend the use of active suction (or negative pressure) to reduce the time required for the particle concentrations within the enclosure to decrease from their initial concentration.

A section in the discussion section has been added to better clarify the risk posed by this leakage and stress the intention that this enclosure is meant as an additional layer of PPE, not a replacement for PPE. The net objective of this enclosure is to reduce risk, not eliminate it. We feel the added lines and existing uncertainties in our measurements make plain that some risk is still present while still adding a layer of protection. The purpose of this enclosure is not to achieve disinfection. Once infectious aerosols contact a surface they are removed from the air and are no longer an aerosol. Particle resuspension from surfaces is quite uncommon unless high flow velocities are applied. The surfaces of the enclosure should be disinfected after use and the enclosure was designed to facilitate this (no internal creases that inhibit disinfection).

Text changes in Discussion:

“The enclosure with two layers of covers applied over the front opening with no suction applied should remain over the patient for at least 15 minutes after the last particle generation event to achieve 90% reduction in particle concentrations within the enclosure”

“However, due to the risk posed by lingering or escaped particles, current standards of PPE should be maintained whenever possible.”

Text changes in Supplemental:

“Particle loss rates are largely dictated by particle size. The suggested 15–20-minute timeframe is enough for all particles 5 µm and larger to gravitationally settle within the enclosure. 50-90% of particles in the 3-5 µm range should also have time to settle within this time frame. Particles on the smaller end are lost primarily through coagulation with larger particles and wall loss due to diffusion and electrostatic attraction. The intermediary sized particles (300 – 2000 nm) are generally the hardest to account for. Some of them will be lost via the various mechanisms described above, and some will be lost due to leakage out of the openings and gaps in the enclosure. Suction of air inside the enclosure will be the most reliable method for fast and near total removal of these particles, such as using a vacuum line inserted through the side hole of the enclosure. However, even without suction, the enclosure will still reduce net exposure to all particle sizes of aerosol produced inside the enclosure in the outside environment surrounding the enclosure.”
